# Spatial variation in avian bill size is associated with temperature extremes in a major radiation of Australian passerines

**DOI:** 10.1098/rspb.2023.2480

**Published:** 2024-01-24

**Authors:** Kalya Subasinghe, Matthew R. E. Symonds, Suzanne M. Prober, Timothée Bonnet, Kristen J. Williams, Chris Ware, Janet L. Gardner

**Affiliations:** ^1^ CSIRO Environment, GPO Box 1700, Canberra, Australian Capital Territory 2601, Australia; ^2^ Research School of Biology, Australian National University, Canberra, Australian Capital Territory 0200, Australia; ^3^ Department of Zoology and Environmental Management, University of Kelaniya, Kelaniya 11600, Sri Lanka; ^4^ Centre for Integrative Ecology, School of Life and Environmental Sciences, Deakin University, Burwood, Victoria 3125, Australia; ^5^ Centre d'Etudes Biologiques de Chizé UMR 7372 Université de la Rochelle-CNRS, 405 route de Prissé la Charrière 79360 Villiers en Bois, France; ^6^ CSIRO Environment, University of Tasmania, College Road, Sandy Bay Tas 7005, Australia

**Keywords:** bill size, climate extremes, Meliphagides, thermoregulation

## Abstract

Morphology is integral to body temperature regulation. Recent advances in understanding of thermal physiology suggest a role of the avian bill in thermoregulation. To explore the adaptive significance of bill size for thermoregulation we characterized relationships between bill size and climate extremes. Most previous studies focused on climate means, ignoring frequencies of extremes, and do not reflect thermoregulatory costs experienced over shorter time scales. Using 79 species (9847 museum specimens), we explore how bill size variation is associated with temperature extremes in a large and diverse radiation of Australasian birds, Meliphagides, testing a series of predictions. Overall, across the continent, bill size variation was associated with both climate extremes and means and was most strongly associated with winter temperatures; associations at the level of climate zones differed from continent-wide associations and were complex, yet consistent with physiology and a thermoregulatory role for avian bills. Responses to high summer temperatures were nonlinear suggesting they may be difficult to detect in large-scale continental analyses using previous methodologies. We provide strong evidence that climate extremes have contributed to the evolution of bill morphology in relation to thermoregulation and show the importance of including extremes to understand fine-scale trait variation across space.

## 1. Introduction

Morphological traits are integral in regulating animal body temperature and maintenance of homeostasis, so greater understanding of climate-related variation in traits may provide insights into adaptation to climate change. Bill size and shape in birds has conventionally been considered in relation to diet and foraging strategies [1] but recent advances in our understanding of thermal physiology suggest an additional role in thermoregulation [2–4]. Bills form part of a network of cranial vasculature and in hot conditions birds can increase blood flow to the highly vascularized and uninsulated bill to increase heat loss [5]. Conversely, at low temperatures heat loss via the bill surface is regulated through vasoconstriction [6]. The larger the bill the greater the potential for heat exchange, so larger bills may be advantageous in hot conditions but a liability at low temperatures [7]. Evidently, bill morphology has direct significance for thermoregulatory function, and therefore may have contributed to the evolution of bill size and shape, with implications for adaptation to climate change.

Like most endotherms, birds maintain their body temperature within a narrow thermal range [8]. Excess metabolic heat is dissipated via evaporative, radiative, convective or conductive mechanisms to maintain body temperature within safe limits [9]. Recent studies have confirmed that a significant portion of body heat is released from the bill surface via radiation at high environmental temperatures, reducing the need for evaporative cooling [2,3,10]. In song sparrows, nearly 10% of metabolic heat production can be released from a bill which accounts for only 2% of total body surface area [7]. Toco toucans (*Ramphastos toco*) release up to 60% of total body heat through the bill [2]. In cold conditions, vasoconstriction can act to significantly limit heat loss from bills; in white Pekin ducks (*Anas platyrhynchos*), 85–117% of total metabolic heat is released from vasodilated bill at 0°C, whereas only 18–25% is lost from a vasoconstricted bill [6].

Observed geographical patterns of bill size are mostly consistent with Allen's rule [11], that the relative size of appendages (mammalian limbs and ears, avian bills and tarsi) changes with latitude reflecting responses to variation in local climate [12]. Larger appendages for a given body size are typical of individuals in lower latitudes where climates are warmer, while smaller appendages are more likely to occur in the colder climates of higher latitudes, helping to conserve body heat by minimizing heat loss [11,13]. A similar pattern is also observed across altitudinal gradients, with larger appendages at lower elevations typified by comparatively warmer environments [14,15]. A study of 214 bird species across several continents showed a significant positive association between bill size and mean annual temperature, with larger bills found in warmer climates [16]. The bill sizes of 10 subspecies of tidal marsh sparrows in California, starlings (*Sturnus vulgaris*) and several species of parrots in Australia, were larger in environments with higher mean maximum summer temperature [7,17,18]. This suggests that bill size patterns may have evolved to improve heat dissipation in warm periods of the year, rather than to conserve heat during cold periods. However, bill sizes of song sparrows (*Melospiza melodia*) in eastern North America, multiple species of Meliphagides in Australia and Oriental magpies (*Pica serica*) in Mainland China showed significant associations with winter temperatures, revealing that Allen's rule patterns may be driven by cold winter temperatures [19–21]. Thus, those aspects of temperature which underlie geographical patterns in bill size can vary.

There are limits to the effectiveness of any thermoregulatory strategy when climate extremes reach or exceed physiological thresholds, resulting in sub-lethal costs and even mortality. Most previous studies of the relationship between bill size and climate have focused on mean annual or mean seasonal temperatures, ignoring the frequency and intensity of temperature extremes and interacting effects of other climate variables. Mean values for temperatures, including mean summer maximum and mean winter minimum temperatures, do not reflect thermoregulatory costs that may be experienced by an individual over a shorter time scale. For example, a mean maximum summer temperature of 30°C may be characterized by daily maxima between 28°C and 32°C or maxima as low as 25°C and higher than 40°C, two regimes with obviously different thermoregulatory costs. When environmental temperatures exceed body temperature (approx. 40°C), the bill will absorb heat from the environment rather than dissipate it, so large bills may be maladaptive in climates where summer temperatures regularly exceed body temperature [22]. For example, although relative bill size in song sparrows increased monotonically with ambient temperature across their range, bill size decreased in parts of the range where average summer temperatures exceeded body temperature [23]. Thus, we predict nonlinear responses to increasing exposure to high temperature extremes if heat exchange strongly underlies bill size variation across space. By contrast, responses to winter temperatures are predicted to be linear, the colder the temperature the smaller the bill size, with no reversal in bill size response, based on thermal performance curves (see [24]).

Temperature will also interact with other climate variables to influence thermoregulatory costs. Humidity has a strong influence on evaporative heat loss: high humidity substantially reduces the efficiency of evaporative cooling [25], so favouring radiative and convective heat loss via the bill. In sociable weavers *Philetairus socius*, for example, high humidity inhibited rates of evaporative water loss by up to 36% at 48°C [25]. Accordingly, larger bills may be a particularly important adaptation in hot, humid environments, because the efficiency of radiative heat loss is unaffected by humidity [7]. Aridity may also increase the cost of evaporative cooling, as its prolonged use may lead to lethal dehydration if body water pools are not replenished via drinking or dietary sources [22]. Larger bills may also therefore be favoured in hot, arid environments where water availability is limited in summer, thereby improving water conservation [23]. However, in cold climates where rainfall is high wet plumage may compromise thermal insulation [26,27], potentially favouring smaller bills that reduce heat loss.

We thus argue that it is crucial to clarify associations between bill size and the frequencies of climate extremes associated with physiological performance and fitness in order to understand the significance of thermoregulatory mechanisms as drivers of bill size variation. Further, failure to control for nonlinear effects of temperature extremes in summer will probably lead to an underestimation of summer effects or miss them entirely, particularly in large-scale spatial analyses. This is because such analyses will be biased by sampling; increasing bill size in warming milder summer climates may be offset by declining bill size in warming hot summer climates. Thus, the methods used by previous studies may have limited capacity to test thermoregulatory mechanisms as drivers of bill size variation. Our recent study highlighted the importance of temperature extremes in survival patterns for two bird communities based on over 37 years of monitoring in semi-arid Australia. The study showed that survival probability declined strongly with increasing exposure to days with thermal maxima greater than 38°C or minima less than 0°C [28]. Thus, we expect strong selection on bill size in its role for thermal physiology.

Here we explore how bill size variation is associated with temperature extremes and how interactions among climate variables influence bill size patterns in a large and diverse radiation of Australasian birds, the Meliphagides. We first describe geographical patterns of bill size in multiple species at a continental scale, then investigate whether spatial patterns are likely to be shaped by the role of the bill in thermoregulation by studying bill size associations with high and low temperature extremes. We used morphological data from 9847 individual museum specimens of 79 species, sampled across the entire geographical ranges of each species, along with the location-specific geographical and climate data to test the following predictions based on thermal physiology:

(1) Relative bill size will decline with increasing exposure to cold extremes to facilitate heat retention.
(2) Relative bill size will vary nonlinearly with hot extremes, specifically:
  Larger bills will be associated with an increase in the frequency of hot extremes. However, in climates where environmental temperature regularly exceeds body temperature, selection pressure acting on bill size will be reversed, favouring smaller bills to reduce heat gain from the environment. Hence, we predict a nonlinear response to extreme high temperatures.
(3) The effects of extreme temperatures will vary with humidity and rainfall, specifically:
  (a) Smaller bills will be found in cold environments with high winter rainfall to compensate for additional heat loss due to reduced thermal resistance of wet plumage.
  (b) Larger bills will be found in hot environments with higher humidity to compensate for the reduced effectiveness of evaporative cooling, and also in hot environments with lower rainfall to mitigate the effects of water loss associated with evaporative cooling. These effects will be greatest where ambient temperature does not regularly exceed body temperature.

## 2. Methods

### (a) Study system

We used 79 species from the infraorder Meliphagides, the largest radiation of Australian passerines (formerly Meliphagoidea; see electronic supplementary material for details), to assess bill size patterns across geographical space and to test for associations between bill size and climate across the Australian continent (electronic supplementary material, table S1).

### (b) Data collection

#### (i) Bird data

We measured bill dimensions (length, width and depth) to estimate bill surface area of 9847 specimens collected between 1956 and 2015 (see electronic supplementary material).

We compiled measurements for the flattened wing chord from the carpal joint to the tip of the longest primary feather, and associated metadata (sex, capture date and capture location [latitude, longitude]) for each specimen from Gardner *et al*. [29]. We collated feeding guild information from the literature, classifying species based on their primary source of food (nectarivore or insectivore). We defined the climate zone from each individual's capture location, as tropical, temperate or arid using the Köppen–Geiger climate classification [30–32] (electronic supplementary material, figure S1).

#### (ii) Climate data

We extracted location-specific climate data, at the capture location of each specimen, for the 72 months prior to the capture date of each bird following Gardner *et al*. [33], at a resolution of 5 km^2^ from the Australian Bureau of Meteorology's daily gridded spatial climate datasets (http://www.bom.gov.au/climate/austmaps/metadata-daily-temperature.shtml; http://www.bom.gov.au/climate/austmaps/metadata-daily-rainfall.shtml), to obtain information on the weather experienced by each individual during five preceding seasons of summer/ winter/ wet/ dry as required. Climate variables included the number of days of thermal maxima (≥35°C, ≥40°C) and thermal minima (<5°C, <0°C), mean summer daily maximum temperature, mean winter daily minimum temperature, total rainfall and mean vapour pressure (see electronic supplementary material for details).

#### (iii) Geographical data

In addition to latitude and longitude, we also collated data for elevation and minimum distance to the coast (GEODATA 9sec DEM; Geoscience Australia [34]) as these variables can influence local climate variation. We also gathered data on IBRA7 (the classification of Australian ecoregions known as bioregions; [35]), corresponding to capture locations, which define 89 geographically distinct regions based on similarity in climate, geology, landforms and vegetation [36].

### (c) Statistical analysis

Data analysis involved the following steps. First, we analysed patterns of bill size across geographical space. Then we tested for associations between bill size and climate at two scales: at a continental level, and then within each climate zone separately (tropical, arid, temperate). Finally, we incorporated phylogenetic information to estimate the extent to which differences among species were phylogenetically determined, first of bill size itself and then species' responses to climatic variation (i.e. the degree to which phylogeny predicts the relationship between bill size and temperature extremes). All analyses were carried out using a Bayesian Markov chain Monte Carlo phylogenetically controlled generalized linear mixed model approach, using the package *MCMCglmm* v. 2.27 [37] in R v. 4.0.3. The global Phylogeny of Birds (www.birdtree.org) was used to construct phylogeny [38]. We downloaded 1000 trees with the ‘Hackett backbone’ [39] and calculated a 50% majority-rule consensus phylogeny using *consensus* function of the *ape* package in R [40]. In all models, bill size and structural body size were log transformed to achieve normality. Both the response and explanatory variables as well as all continuous control variables except the year of capture were z-standardized before fitting the model. The year of capture was centred on its mean (1985). All models included a phylogenetic matrix of the species as a random effect. We assessed model convergence using visual diagnostics (trace, density and autocorrelation plots electronic supplementary material, figure S2), in particular using Bayesplot [41]. Effective sample sizes were above 800.

#### (i) Bill size patterns across geographical space

We described broad-scale geographical patterns of bill size across Meliphagides by fitting a model [m1], with bill size as the response variable and geographical variables (i.e. latitude, longitude, elevation, minimum distance to coast) as explanatory variables, while controlling for structural body size (using wing length as the proxy), feeding guild, sex, season and year of capture as fixed effects, and IBRA region and species as random effects (see electronic supplementary material for details).

Three distinctly different environments are found along the latitudinal gradient within Australia: humid tropics at northernmost latitudes, hot and dry arid zone at mid latitudes and cold temperate zone at southernmost latitudes. Bill size might therefore not change linearly with latitude due to differences in thermoregulatory costs between climate zones. Hence, to further explore the pattern with latitude, we fitted a separate model [m2] including a quadratic term of standardized absolute latitude as an explanatory variable (see electronic supplementary material for [m1, m2]). We ran the models with 13 000 iterations using weakly informative priors, with a burn-in of 3000 and thinning interval of 10. We applied default broad Gaussian priors for fixed effects and inverse-Wishart priors, with parameters V = 1 and nu = 0.002, for random effects.

#### (ii) Testing associations with climate

After describing geographical patterns, we investigated the importance of including climate extremes in models and the nature of the relationship between bill size and climate extremes. We started by looking at associations at the continent scale. We fitted a model [m3] using *MCMCglmm* as described earlier and model selection to identify the most parsimonious model (referred to as the best model) based on Akaike information criterion corrected for small sample sizes (AICc) using *MuMIn* package v. 1.43.17 [42] (see electronic supplementary material for [m3]).

We included all climate variables required to test our hypotheses i.e. days ≥35°C, ≥40°C, <5°C, <0°C, mean maximum summer temperature, mean minimum winter temperature, mean summer vapour pressure, total summer rainfall, total winter rainfall in the model, along with the quadratic terms for days ≥35°C, ≥40°C, <5°C, <0°C (i.e. to account for the likelihood that bill size might increase up to a certain level of exposure to hot extremes and decrease at very high exposures to minimize heat gain), mean maximum summer temperature, mean minimum winter temperature, and mean summer vapour pressure as well as the interactions between; mean summer vapour pressure with days ≥35°C, ≥40°C and mean maximum summer temperature, total summer rainfall with days ≥35°C, ≥40°C and mean maximum summer temperature, and total winter rainfall with days <5°C, <0°C and mean minimum winter temperature in global model (see model m3).

When performing model selection for m3, the subsets that included both days ≥35°C and ≥40°C or days <5°C and <0°C were excluded from the final model set. We tested two values for each extreme in order to identify likely threshold for bill size responses. Note that the subsets with hot extremes (days ≥35°C or ≥40°C) also included mean maximum summer temperature and cold extremes (days <5°C or <0°C) mean minimum winter temperature. This was done to disentangle the effects of mean seasonal temperatures (maxima/minima) from those of extreme days *per se* [43–45]. This approach follows [28]. All climate variables were z-standardized prior to analysis. Other than climate variables, we included all control variables in m1 and m2.

Following analyses of continent-wide associations between bill size and climate, we ran separate analyses for each climate zone. Here, we created model subsets for m3 separately for each climate zone to identify any specific climate conditions that might underlie size patterns.

#### (iii) Testing the effect of phylogeny and species variation in bill size response

We quantified the degree of phylogenetic signal in bill size independent of body size and climate across Meliphagides, i.e. how much variation in bill size is explained by shared ancestry [46]. We also tested if there were differences among species in how they responded to climate extremes within each climate zone (see electronic supplementary material for details).

## 3. Results

Bill size (surface area) of Meliphagides ranged between 18.11 mm^2^ in the weebill *Smicrornis brevirostris* and 508.18 mm^2^ in the blue-faced honeyeater *Entomyzon cyanotis*. Males of Meliphagides had significantly larger bills, relative to body size, than females, and individuals captured during winter had relatively smaller bills than in other seasons (electronic supplementary material, table S2). The latter confirmed a seasonal effect on bill size associated with bill wear, as indicated in previous studies [47]. Feeding guild, however, showed no significant association with relative bill size (electronic supplementary material, table S2).

### (a) Bill size patterns across geographical space

**Figure 1.**
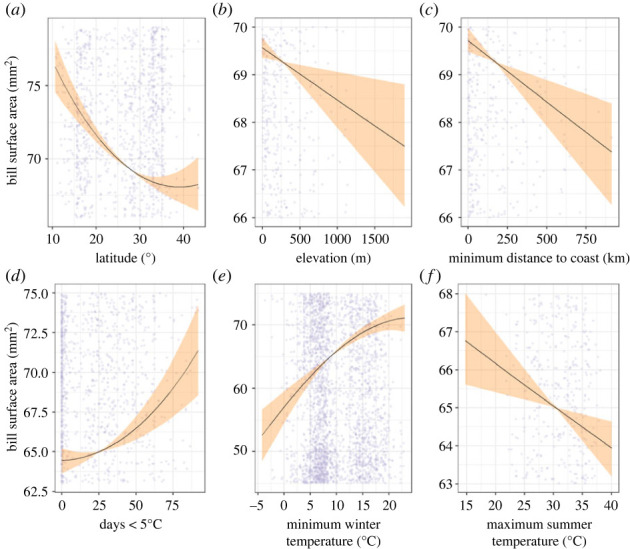
Relationships between relative bill size and geographical variables (*a*) latitude, (*b*) elevation, (*c*) minimum distance to coast, and climate variables (*d*) days < 5°C, (*e*) mean minimum winter temperature, (*f*) mean maximum summer temperature, across the continent. Shaded area shows 95% credibility intervals around fitted values, when all other predictors (including their parameter estimates) are held constant for (*a,b,c*) and for (*d,e,f*) when posterior mean of non-focal parameters used for predictions. (Electronic supplementary material, figure S6 shows all data points.)

There was a significant relationship between latitude and relative bill size across species after controlling for phylogeny (electronic supplementary material, table S2). Bill size was larger on average in the tropical north of the continent compared with the temperate south which is characterized by colder climates, a spatial pattern conforming to Allen's rule. A decrease in AICc value (from −9727.019 to −9730.772; electronic supplementary material, table S2) with the inclusion of a quadratic term for latitude indicated that the relationship with latitude is nonlinear; bill size decreased strongly before levelling off at southernmost latitudes (figure 1*a*). Bill size variation across latitude did not differ significantly between feeding guilds (guild × latitude interaction; electronic supplementary material, table S2). There were significant negative associations with elevation and minimum distance to coast (figure 1*b,c*; electronic supplementary material, table S2).

### (b) Associations between bill size and climate at a continent level

The best model of the subset of 26 113 models generated in the continent-wide analysis indicated that bill size variation was best explained by the frequency of days < 5°C, mean minimum winter temperature and mean maximum summer temperature with an AICc of −9765.50 (electronic supplementary material, table S3). The best model with no climate extremes ranked 126 with an AICc of −9757.30. In the best model that included extremes, mean minimum winter temperature showed a strong positive association with relative bill size, with the largest effect size of any predictor after wing length (figure 1*e*, table 1). The effect sizes of the unexpected positive association between bill size and days < 5°C and the negative association with mean maximum summer temperature were lower (figure 1*f*, table 1).

**Table 1.** Parameter estimates for the best model for analysis of relative bill size in the continent-wide analysis and associated credibility intervals (CrIs). The estimates for climate variables, where 95% CrIs do not contain zero are highlighted in italics. All continuous variables except year of capture are standardized.

	parameter	estimate	95% CI
continent	intercept	−0.169	−1.788; 1.447
	days <5°C	*0.023*	*0.009; 0.040*
	days <5°C ^2	*0.010*	*−0.004; 0.015*
	min. winter temperature	*0.085*	*0.063; 0.106*
	min. winter temperature ^2	*−0.013*	*−0.022; −0.004*
	max. summer temperature	*−0.012*	*−0.020; −0.004*
	year of capture	*−0.0003*	*−0.0007; 0.0004*
	Sex		
	male	*0.051*	*0.045; 0.059*
	Season		
	spring	*0.009*	*0.001; 0.017*
	summer	*−0.021*	*−0.011; −0.033*
	winter	*−0.017*	*−0.026; −0.009*
	wing length	*0.440*	*0.414; 0.465*

### (c) Bill size associations in each climate zone

#### (i) Climatic variation

There was considerable variation in the climatic conditions in the different climate zones (electronic supplementary material, figure S3). Tropical populations experienced high humidity (mean = 32.31 hPa ± 2.46 s.d.) and high rainfall (mean monthly total rainfall = 199.59 mm ± 63.92 s.d.) during the wet season which co-occurred with high frequencies of days ≥35°C.

Arid populations also experienced high frequencies of days ≥35°C in summer, including severe extremes (days ≥40°C), but in low rainfall conditions (mean monthly total rainfall = 60.80 ± 63.36 s.d.) (electronic supplementary material, figure S3). Most populations across the temperate zone experienced low humidity (mean = 24.85 ± 4.37 s.d.) and rainfall (mean monthly total rainfall = 95.45 mm ± 77.99 s.d.) in summer, and fewer hot extremes than populations in either the tropical or arid zones. Exposure to cold conditions <5°C during winter was common across both temperate and arid zones.

#### (ii) Associations with climate extremes

Bill size variation was best explained when extremes were included in the model, except in the tropical zone (but see below), as indicated by the poorer AICc value of the best model without extremes (temperate zone: ΔAICc of model without extremes = 13.11; arid zone: ΔAICc of model without extremes = 0.62; tropical zone: ΔAICc of model *with* extremes = 1.63)

Relative bill size in the arid zone was associated with the frequency of cold extremes <5°C, whereas in temperate regions hot extremes ≥35°C were important (table 2). Further, the effect of temperature extremes on bill size was mediated by humidity and rainfall. The parameter estimates for the best model of each of the three climate zone analyses and associated credible intervals (CrIs) are given in table 2. All models with ΔAICc ≤ 2 for all climate zone analyses are shown in electronic supplementary material, table S4–S6.

**Table 2.** Parameter estimates for the top model for arid and temperate zones, and the second-best model (the only model with climate variables among all models with ΔAICc ≤ 2) for the tropical analysis, and associated CrIs. The estimates for climate variables, where 95% CrIs do not contain zero are highlighted in italics. All continuous variables except year of capture are standardized.

	parameter	estimate	95% CI
arid zone	intercept	−0.079	−2.092; 1.787
	days <5°C	*0.042*	*0.008; 0.070*
	days <5°C ^2	0.010	−0.001; 0.019
	min. winter temperature	*0.084*	*0.034; 0.130*
	min. winter temperature ^2	*−0.024*	*−0.041; −0.007*
	mean summer vapour pressure	0.012	−0.002; 0.025
	winter rainfall	−0.001	−0.015; 0.011
	year of capture	−0.001	−0.001; 0.000
	sex		
	male	0.052	0.039; 0.062
	season		
	spring	0.017	0.004; 0.031
	summer	0.034	0.009; 0.053
	winter	−0.028	−0.041; −0.012
	wing length	0.395	0.355; 0.435
	days <5°C: winter rainfall	*−0.038*	*−0.056; −0.021*
	min. winter temperature: winter rainfall	*−0.060*	*−0.088; −0.037*
temperate zone	intercept	−0.171	−1.673; 1.367
	days ≥35°C	0.002	−0.018; 0.023
	summer rainfall	−0.007	−0.021; 0.008
	summer vapour pressure	*0.044*	*0.021; 0.068*
	summer vapour pressure ^2	−0.030	−0.058; 0.002
	max. summer temperature	*−0.044*	*−0.069; −0.017*
	max. summer temperature^2	*−0.051*	*−0.082; −0.018*
	year of capture	0.000	0; 0.001
	sex		
	male	0.054	0.044; 0.065
	season		
	spring	0.001	−0.013; 0.013
	summer	0.009	−0.006; 0.023
	winter	−0.020	−0.034; −0.007
	wing length	0.467	0.430; 0.505
	days ≥35°C: summer vapour pressure	*−0.013*	*−0.023; −0.006*
	days ≥35°C: summer rainfall	*−0.031*	*−0.046; −0.015*
	summer rainfall: max. summer temperature	0.005	−0.007; 0.016
	summer vapour pressure: max. summer temperature	*0.075*	*0.015; 0.138*
tropical zone	intercept	−0.135	−1.171; 1.339
	max. summer temperature	−0.009	−0.035; 0.018
	wet season vapour pressure	0.004	−0.020; 0.028
	wet season vapour pressure ^2	*−0.043*	*−0.071; −0.019*
	year of capture	−0.002	−0.002; −0.001
	sex		
	male	0.030	0.010; 0.049
	season		
	wet season	0.002	−0.020; 0.026
	wing length	0.584	0.503; 0.658
	max. summer temperature: wet season vapour pressure	*0.043*	*0.018; 0.071*

**Figure 2.**
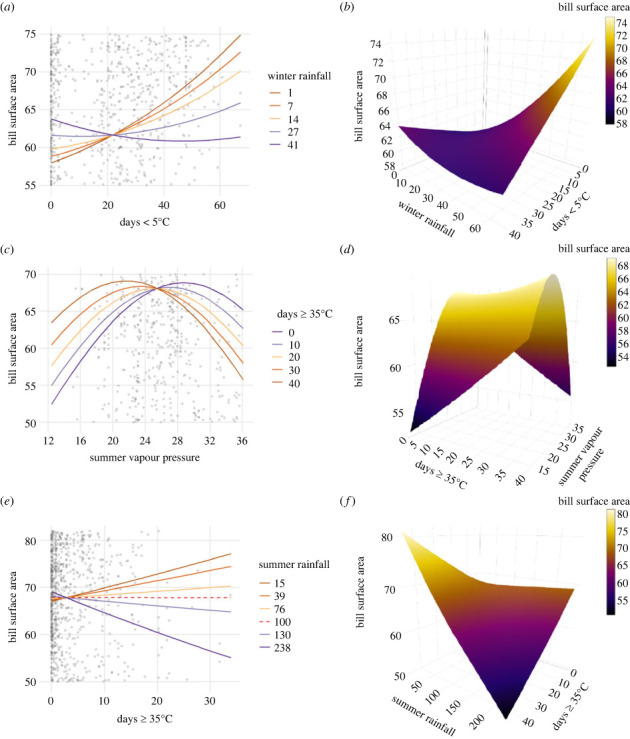
Associations between relative bill size and frequency of extreme temperatures within climate zones. Shown are relationships between bill size and (*a*) days <5°C in interaction with winter rainfall (mm) at five quantiles, 5th, 25th, 50th, 75th and 95th across 50 species within the arid zone and (*b*) corresponding three-dimensional representation; (*c*) frequency of days ≥35°C in interaction with mean summer vapour pressure (hPa) at five quantiles across 57 species within the temperate zone and (*d*) corresponding three-dimensional representation; (*e*) frequency of days ≥35°C in interaction with summer rainfall (mm) at five quantiles across 57 species within the temperate zone and (*f*) corresponding three-dimensional representation. Red-dash line in (*e*) shows the amount of rainfall above which bill size shows an increase with increasing hot extremes. (Electronic supplementary material, figure S7 shows all data points.)

*Arid zone*. We found no strong evidence for direct linear or nonlinear associations between relative bill size and the frequency of hot extremes, days ≥35°C (or ≥40°C electronic supplementary material, table S4), as neither the linear nor quadratic terms were included in the best model (table 2). Similarly, summer humidity was not included in best model (table 2). The relationship between bill size and the frequency of exposure to daily minima < 5°C, however, was significant and this association was mediated by winter rainfall: bill size declined with increasing exposure to days <5°C, but only in high rainfall conditions (table 2, days <5°C x winter rainfall interaction; figure 2*a,b*). The association between bill size and mean minimum winter temperature was also significant with slightly smaller effect sizes (table 2). This relationship was also mediated by winter rainfall (table 2, mean minimum winter temperature x winter rainfall interaction) with a strong reduction in bill size in warmer winters as rainfall increased (electronic supplementary material, figure S4a, b).

*Temperate zone*. There were no associations between relative bill size and exposure to cold extremes <5°C (or 0°C electronic supplementary material, table S5) as these variables were not included in the best model (table 2). By contrast, relative bill size increased with increasing summer humidity (mean summer vapour pressure) up to a point, then decreased. This relationship was mediated by temperature extremes in summer (summer vapour pressure × days ≥35°C interaction, table 2, figure 2*c*,*d*), such that at low humidity, bill size increased with increasing exposure to days ≥35°C. As humidity increased, there was a switch toward smaller bill size with increasing exposure to days ≥35°C.

The association between bill size and mean summer maximum temperature was nonlinear (table 2, max. summer temp.^2^), and there was an interaction with humidity (max. summer temperature × summer vapour pressure). The association between mean maximum summer temperature and humidity indicated a switch towards larger bill size at higher humidity in hotter environments (electronic supplementary material, figure S4c,d).

There was also a significant interaction between days ≥35°C and summer rainfall (summer rainfall × days ≥35°C interaction, table 2, figure 2*e,f*). Bill size increased with increasing exposure to days ≥35°C in climates when average summer rainfall is <100 mm (figure 2*e,f*).

*Tropical zone*. We did not consider cold extremes as there were very few populations that experienced these conditions (electronic supplementary material, figure S3). The best model contained only wing length, sex and year of capture. However, the second-best model was equally favoured (ΔAICc = 0.362) and indicated that wet season vapour pressure is associated with bill size variation and this relationship is mediated by mean maximum summer temperature (electronic supplementary material, figure S4e,f; table 2). As humidity increases, there was a switch towards larger bills at higher mean maximum summer temperatures, a pattern that was also observed across temperate populations with mean maximum summer temperature (electronic supplementary material, figure S4e,f).

### (d) Evolutionary history, climate extremes and bill size variation

There was a strong effect of phylogeny (*λ* of 0.895, 95% CrI = 0.853–0.935) on bill size, independent of body size, across species of Meliphagides (electronic supplementary material, figure S5). Thus, more closely related species have similar relative bill sizes, (see phylogenetic tree, electronic supplementary material, figure S5).

**Figure 3.**
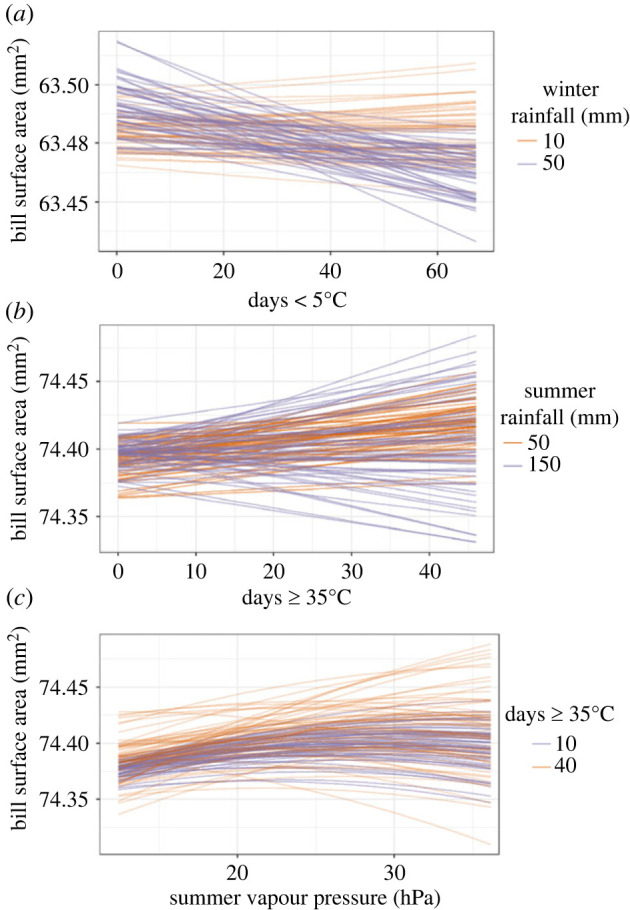
Species variation in the bill size response to extreme temperatures. Shown are bill size associations with (*a*) frequency of exposure to daily minima < 5°C in arid zone birds at 10 mm (orange) and 50 mm (blue lines) of winter rainfall, (*b*) frequency of exposure to daily maxima ≥35°C in temperate zone birds at 50 mm (orange lines) and 100 mm (blue lines) of summer rainfall and (*c*) summer vapour pressure in temperate zone birds when exposure to daily maxima ≥35°C is 10 (blue lines) or 40 days (orange lines) in summer. Each line (orange or blue) presents the model-predicted mean bill size of individual species after controlling for body size, sex, year of capture and IBRA region.

A substantial improvement in AICc was noted after inclusion of a random slope for the non-phylogenetic species term (m5), in addition to a random intercept (ΔAICc of model without the species term arid zone = 140.18; temperate zone: 255.74; electronic supplementary material, table S7), indicating that the associations we observed between bill size and climate probably differ among species. However, when comparing responses among species (figure 3), there were similarities in bill size patterns across species for those experiencing similar conditions. In the arid zone, the association between bill size and the frequency of daily minima below 5°C is relatively similar between species, for individuals experiencing high rainfall (figure 3*a*). Likewise in the temperate zone, the association between bill size and the frequency of daily maxima above 35°C is similar in low rainfall environments, whereby the majority of species showed an increase in bill size with increasing exposure to hot extremes (figure 3*b*). The bill size pattern with humidity was relatively similar at both exposures, i.e. 10 and 40 days with temperatures ≥35°C) (figure 3*c*). There was no obvious reduction in AICc with addition of random slopes to the phylogenetic species term to m5 for both arid and temperate models (m6; electronic supplementary material, table S7), indicating that phylogeny has no effect on species responses to climate extremes in these analyses.

## 4. Discussion

We examined the relationship between bill size and climate extremes in our correlative study that explores the adaptive significance of bill size in relation to its role in thermoregulation in Meliphagides. Our results are robust to the inclusion of the correlated effects of mean temperatures, are based on comprehensive specimen sampling and match climate and morphology at appropriate spatial and temporal scales. Overall, across the continent, bill size variation was associated with both climate extremes and climate averages. Bill size was most strongly associated with average minimum winter temperature with smaller bills in environments with colder winters. However, the nature of the associations with temperature extremes varied among climate zones, were moderated by rainfall and humidity and in the case of summer temperatures were nonlinear. There was a strong effect of phylogeny on bill size across Meliphagides species but there was no phylogenetic signal for associations between bill size and climate.

### (a) Patterns of bill size are consistent with Allen's rule

Our study, based on 9847 individuals, provides strong evidence that bird bills conform to Allen's rule at a continental scale in Southern Hemisphere passerines. In particular, Meliphagides inhabiting northerly latitudes closer to the equator displayed relatively larger bills than those in more southerly latitudes. We also detected a nonlinear relationship between bill size and latitude, with a reduction in the rate of change evident at higher latitudes. Such inconsistency implies that local climate underlies the bill size association with latitude. Elevation can confound bill size patterns across latitude by creating a vertical climate gradient [14]. Here, we report a negative relationship between bill size and elevation in line with previous studies, confirming that Allen's rule applies to both latitudinal and elevational gradients [16]. In addition to latitude and elevation, we noted a significant negative (although smaller) association between bill size and direct distance to the coastline, as observed elsewhere [48].

The strong associations between bill size and climate across space suggests that the latitude effect characteristic of Allen's rule is a proxy for climate, as is widely accepted [16]. Winter conditions, including both minimum winter temperature and exposure to winter extremes (days <5°C), best explained bill size variation across the continent. Among climatic predictors the association with mean minimum winter temperature showed the largest effect size, with smaller bills associated with lower winter minimum temperatures. Our results concur with the continent-wide analysis of bill size in Australian Meliphagides by Friedman *et al*. [20] who found that bill size variation was strongly associated with minimum winter temperature. However, our more comprehensive analyses sampling multiple populations from across the entirety of each species' range suggest that continent-wide analyses obscure the significant role of local climatic regimes in driving bill size variation.

### (b) Bill size and cold extremes (prediction 1)

Our study indicated a significant *positive* association between bill size and increasing frequency of days <5°C in the continental analysis, but this pattern occurs after already controlling for minimum winter temperature. Overall, bill size is smaller than average in environments with increasing frequency of extreme cold days <5°C, but not as small as expected given average coldness of the local climate. Consequently, upon controlling for average minimum temperature, relative bill size is negatively associated with more cold extreme days, consistent with prediction 1.

In the climate zone analyses, the frequency of exposure to cold extremes was associated with bill size variation only in the arid zone. Here, bill size reduction was associated with increasing exposure to days <5°C only in environments with high winter rainfall (see below for discussion). There was no effect of cold extremes in the temperate zone, despite our expectations. Although both arid and temperate zone populations experienced similarly severe cold winters, a greater proportion of populations in the arid zone were subject to such conditions compared with the temperate zone, at least in our sample (see electronic supplementary material, figure S3). Perhaps this reflects the large latitudinal range of the temperate zone classification we used, obscuring any effect of cold extremes in our temperate analysis. Alternatively, this result might reflect a trade-off between optimal bill sizes in different seasons, with stronger selection for larger bills in summer.

### (c) Bill size and hot extremes (prediction 2)

Our continent-wide analysis showed that bill size decreased with increasing maximum summer temperature, contrary to prediction, and there was no association with hot extremes (days >35° or days >40°C). However, Gardner *et al*. [33] found that the effect of maximum summer temperature on the direction of the bill size response appears to be dependent on its interaction with rainfall or humidity. Therefore, the broad spatial scale of our continental analysis may have obscured the significance of local climatic regimes in driving bill size variation. This highlights the need for caution when interpreting the results of large-scale climate analyses; the overall bill size response to summer temperatures will depend on the relative sampling of mild versus hot summer climates. Moreover, responses to low (winter) temperatures are predicted to be linear, bill size decreasing the colder (or colder and wetter) it gets, until some constraint on size (e.g. effect on foraging) is reached, but there is no temperature-related reversal. Thus, bill size responses to low temperatures may be more easily detected in such analyses (e.g. [20,49]).

In climate zone analyses we found no evidence for a positive association between bill size and high temperature *per se*. Rather, hot extremes (days >35°C) in the temperate zone were associated with bill size variation in interaction with humidity and rainfall (see next section). In these cases, the interactive effect of extreme temperature was nonlinear, with reversal in the response of bill size in line with prediction 2, and similar to that observed by Greenberg and Danner [23] and Gardner *et al*. [33]. Thus, relationships between bill size and hot extremes are complex but consistent with thermal physiology. This result highlights the need for comprehensive sampling of bill morphology and the inclusion of climate extremes to capture the necessary variation at appropriate scales. It suggests that previous studies have limited capacity to test thermoregulatory mechanisms as drivers of bill size variation. For example, Friedman *et al*. [20,49] extracted climate data for every cell in each species’ distribution for the 50 years between 1950 and 2000, then averaged over time and space to provide a set of bioclim variables for each species. While they provided a first-time analysis, it is difficult to imagine how those data relate causally to morphological variation captured on the basis of measuring an average of just five individuals per species. Such apparent mismatch in scale between measurement of climatic variation and morphology at the species level makes it highly unlikely that they would identify the specific climate conditions that drive bill size variation, particularly as physiology predicts nonlinear bill size responses to high temperatures. The limitation of using species averages, and the value of explicitly including intraspecific variation has been discussed elsewhere (e.g. [50]).

In contrast to previous studies, we used climate data (approx. 2.5 arc minutes) from the collection location of each *specimen* in our dataset, for the 5 years prior to the date of collection, to reflect the actual climate (averages and extremes) experienced by each individual, thereby directly matching climate with bill morphology at appropriate spatial and temporal scales. This approach allowed us to more directly test for climatic conditions associated with bill size variation, at the level of the individual (both within species and between species) and identify the summer temperature thresholds at which a reversal in bill size was observed, in line with predictions from physiology.

We did not observe a strong association between bill size and hot extremes in the arid zone where such extremes are common. This was unexpected, given growing evidence for the importance of bills in heat dissipation in hot conditions [2,3,51]. This result might represent a trade-off between optimal bill sizes in different seasons, given the arid zone is characterized by climatic extremes in both winter and summer, and evidence of strong selection for smaller bills in winter in the arid zone. If large bills compromise winter survival, birds may need to rely on other methods to dissipate heat in summer when ambient temperatures are high. Heat loss may also be achieved through non-evaporative heat dissipation via the legs, and Allen's rule predicts increases in leg size in relation to temperature [7]. Our results highlight the need to consider interactions among multiple traits when interpreting patterns of trait size variation and their role in thermal performance.

### (d) Temperature extremes interact with other climate variables (prediction 3)

#### (i) Effects of winter extremes are mediated by rainfall in arid zone birds (prediction 3a)

In the arid zone, the strongest predictor of bill size was exposure to days <5°C and, consistent with our prediction (3a) this association was mediated by winter rainfall. In particular, bill size decreased with increasing exposure to days <5°C but only in environments with relatively high winter rainfall. Despite lower energy intake in winter due to reduced food availability, birds need to increase energy expenditure to maintain optimal body temperatures [52]. Energy requirements for thermoregulation are greater in cold, wet environments where plumage insulation is compromised [26,27], and this is likely to have consequences for fitness. Indeed, Gardner *et al*. [53] found that white-browed scrubwrens (*Sericornis frontalis*) that were exposed to higher frequencies of cold wet days <5°C in winter were less likely to survive. Mechanisms that aid heat conservation, such as smaller bills, may therefore be crucial in such conditions in line with our findings.

#### (ii) Effects of summer extremes are mediated by humidity and rainfall in temperate zone birds (prediction 3b)

We found strong support for prediction 3b, that hot extremes interact with other climate variables to influence bill size patterns. In particular, we found a positive association between bill size and increasing frequency of days >35°C, but only in dry environments where summer rainfall was <100 mm. Our result supports the idea that larger bills may be adaptive in hot, arid environments where water availability is limited in summer due to improvement in water conservation [7].

Similarly, we found a positive association between relative bill size and humidity in summer in the temperate zone (when average days ≥35°C set to mean) with larger bills in more humid environments. Latent heat loss via evaporation becomes less effective as humidity increases, potentially increasing the importance of heat dissipation via radiative structures such as the bill [25,54]. The effect of humidity on bill size was nonlinear and mediated by increasing exposure to hot extremes with a switch toward smaller bills in hotter, humid conditions. Interestingly, the switch towards smaller bills occurred earlier across the humidity gradient in locations with hot summers characterized by many extreme days (approx. 22 hPa at 40 days exposure to days ≥35°C in our sample) and later in locations with relatively cooler summers with fewer extreme days (approx. 27 hPa at 10 days exposure to days ≥35°C), consistent with prediction 2. The value of larger bills that compensate for reduced efficiency of evaporative cooling declines with increasing exposure to hot extremes, because bills become a heat sink when air temperature exceeds body temperature. This may underlie the switch from large to small bills at different frequencies of hot extremes along the humidity gradient. Our findings are consistent with Gardner *et al*. [33] who found that humidity had a much stronger association with bill size than did temperature *per se*, but temperature mediated the association between humidity and bill size.

#### (iii) No effect on bill size of hot extremes associated with humidity in tropics

The best model for tropical zone Meliphagides that included climate variables showed a significant association between bill size and humidity, similar to the temperate zone. Unlike temperate birds, however, there was no switch towards smaller bills with exposure to hot extremes. Perhaps individuals in our sample did not experience the critical temperature threshold necessary for such a switch, even though they were frequently exposed to temperatures >35°C. Indeed, there were few days in our tropical sample with maxima >40°C, when strong effects might be observed. Moreover, tropical birds in our sample were smaller, on average, than temperate individuals (electronic supplementary material, table S8) and therefore likely to be more efficient at dissipating body heat via convection [55]. The advantage of smaller size, together with less exposure to maxima >40°C, might make them less vulnerable to temperature extremes than the temperate individuals [56]. The observed interaction between maximum summer temperature and summer humidity (larger bills in hot environments coinciding with high humidity but no reversal in the bill size response), is consistent with this suggestion.

Overall, our results suggest that bills have an important role in balancing heat budgets, specifically in hot dry, hot humid and cold wet environments. This suggests that shifts between reliance on convective, evaporative or radiative cooling are associated with different climatic regimes, consistent with predictions from thermal physiology.

### (e) Adaptation of bill size to climate: evolutionary versus plastic effects

We found a strong phylogenetic signal for bill size, independent of body size, across Meliphagides species, with more closely related species showing greater similarities in bill size. Such patterns have been interpreted in relation to diet and feeding ecology, although a recent macro-evolutionary study found diet contributed very little (approx. 12%) to variation in bill morphology across multiple lineages [57].

In contrast to the effect of phylogeny on bill size *per se*, associations between relative bill size and climate extremes showed no phylogenetic signal among closely related species. Given the possible associations between phylogenetic signal (species-level heritability) and short-term heritability, this might suggest that observed climate-related bill size patterns occurred independently within species in response to local conditions, although attempts to demonstrate such links have typically not found that that is a clear association [58].

These findings are significant in relation to the question of whether bill size responses to climate are driven by plasticity or microevolution. The size and shape of bills has been shown to be highly heritable [59], although there is some evidence for developmental plasticity [60–62]. We cannot rule out plasticity for patterns we observed. However, the broad ecological, geographical and phylogenetic scale of our study does suggest an adaptive evolutionary response to climate driven by strong selection for the role of bill size in thermal physiology.

## 5. Conclusion

Our study found bill size patterns across Meliphagides conform to Allen's rule across broad geographical space, some 33 degrees of latitude (or *ca* 4000 km). However, we show that some finer scale variation in bill size is obscured in the broader continental-level analysis, demonstrating the importance of considering different climate regimes at sub-continental geographical scales. In particular, the combination of restricted specimen sampling and use of climate averages alone, can make it difficult to detect nonlinear responses to high summer temperatures. Associations between bill size and climate observed at the level of climate zones differ from continent-wide associations yet are consistent with studies relating to physiology and a thermoregulatory role for avian bills. Our results suggest that radiative heat loss via the bill may be particularly useful for birds living in dry climates (rainfall <100 mm) in the temperate zone where use of evaporative cooling is constrained due to limited water availability, and also for birds living in humid environments with fewer days of hot extremes exceeding body temperature. Further, heat loss via bills is a greater concern for birds experiencing cold winters, particularly within the arid zone. We show the importance of including extremes of climate in addition to climate means, and accounting for possible interactions and nonlinearity to better understand fine-scale variation in trait size across space. Our study provides the best evidence to date for the ecological significance of the avian bill in its role as a thermoregulatory organ, and that climate extremes may have contributed to the evolution of bill morphology. If we are to predict species' responses to ongoing climate change, particularly the frequency, intensity and duration of extreme heat, it is necessary to understand underlying physiological and evolutionary mechanisms and associated fine-scale relationships. Our work is a novel methodological advance in this direction.

## Data Availability

Data are available from Dryad Digital Repository: https://doi.org/10.5061/dryad.9zw3r22m1 [63]. Supplementary material is available online [64].
